# TSH receptor antibody as a predictor of difficult robotic thyroidectomy in patients with Graves’ disease

**DOI:** 10.1007/s11701-024-01869-y

**Published:** 2024-03-04

**Authors:** Ja Kyung Lee, Yoon Kong, Jae Bong Choi, Woochul Kim, Hyeong Won Yu, Su-jin Kim, Young Jun Chai, June Young Choi, Kyu Eun Lee

**Affiliations:** 1 Department of Surgery, Seoul National University Bundang Hospital, Seongnam, South Korea; 2 Department of Surgery, Seoul National University Hospital, Seoul, South Korea; 3 Department of Surgery, Seoul National University College of Medicine, Seoul, South Korea; 4 Department of Surgery, Seoul Metropolitan Government Seoul National University Boramae Medical Center, Seoul, South Korea

**Keywords:** Difficult thyroidectomy, Robotic thyroidectomy, Graves’ disease, Thyroid stimulating hormone receptor antibody, Intraoperative blood loss, Operation time

## Abstract

Thyroidectomy in Graves’ disease can be challenging due to greater thyroid size and vascularity. While thyroid stimulating hormone receptor antibody (TRAb) level is associated with disease severity and thyroid vascularity, its impact on operative outcomes remains unclear. This study aimed to compare challenging factors for robotic thyroidectomy (RT) and open thyroidectomy (OT) in Graves’ disease patients, including TRAb as a predictive factor for difficult thyroidectomy. This retrospective study included Graves’ disease patients who underwent total thyroidectomy between September 2013 and January 2023. The clinical characteristics and operative outcomes were compared between patients who received OT and bilateral axillo-breast approach RT. Factors affecting operation time and estimated blood loss (EBL) were evaluated in both groups using regression analyses. A total of 85 patients received either OT (n = 48) or RT (n = 37). Median thyroid volumes in the OT and RT groups were 72.4 g and 57.6 g, respectively. Operation time was affected by thyroid volume in both groups. Additionally, higher thyroid hormone levels and bilateral central neck node dissection prolonged operation time in the RT group. EBL was marginally associated with thyroid volume in the OT group. However, in the RT group, TRAb level was independently associated with greater EBL (p = 0.04), while no significant association was found with thyroid volume. Predictive factors for difficult thyroidectomy differed by operation approaches. TRAb significantly predicted intraoperative bleeding in RT, while this association was absent in OT. Caution is warranted when performing RT on Graves’ disease patients with high TRAb levels.

**Supplementary Information:**

The online version contains supplementary material available at 10.1007/s11701-024-01869-y.

## Introduction

Thyroidectomy for Graves’ disease (GD) can be challenging due to its characteristics, including thyroid enlargement, tissue friability, and hypervascularity [1]. Thus, intraoperative blood loss and the risk of postoperative hematoma are greater in patients with GD than other thyroid diseases [2, 3]. Thyroid hypervascularity in GD arises from increased inflammation and angiogenesis, driven by autoantibodies that stimulate the thyroid stimulating hormone (TSH) receptor (TRAb) [4, 5]. Although TRAb concentration is clinically important and is indicative of GD severity [6], its association with operation outcomes remains unclear.

Recently, robotic thyroidectomy (RT) is increasingly used in operations on patients with more complicated thyroid conditions, including Graves’ disease [7–11]. In fact, the mean thyroid volumes from RT studies of GD patients have steadily increased over 10 years, from 16.6 g in the first study to 86.2 g in a recent report, indicating that large thyroid volume is no longer a contraindication for RT [7–9, 12–16]. These studies, however, did not report the degree of thyroid vascularity in patients with GD, and no study to date has evaluated the association of TRAb with operative outcomes in patients who underwent RT.

This study aimed to evaluate factors that could increase the difficulty of surgery in GD patients, and to determine whether these predictive factors differ in patients undergoing conventional open thyroidectomy (OT) and RT. Moreover, because TRAb is correlated with both GD severity and thyroid vascularity, the associations of TRAb concentrations with outcomes of OT and RT were also evaluated.

## Materials and methods

### Patients

This study retrospectively collected medical records of GD patients who received total thyroidectomy by a surgeon with more than 10 years of clinical experience from September 2013 to January 2023. GD was diagnosed based on clinical and laboratory findings, including elevated triiodothyronine (T3) or free thyroxine (fT4) levels, reduced TSH levels, elevated TRAb, and radioactive iodine uptake or ultrasonography findings suggestive of GD. Patients were excluded if they lacked TRAb information within 6 months prior to surgery, underwent concurrent modified radical neck dissection or operation on another organ, underwent subtotal thyroidectomy, or were on anticoagulant or antiplatelet therapy. Patients were divided into two groups, those who underwent conventional OT and those who underwent bilateral axillo-breast approach (BABA) RT. The institutional review board approved this study (IRB No.: B-2305-831-103) and waived the requirement for informed consent due to the retrospective design.

### Laboratory tests

The most recent thyroid hormone levels and the highest TRAb levels within 6 months prior to surgery were collected. Serum fT4, T3, and TSH concentrations were measured using an Elecsys^®^ electrochemiluminescence immunoassay (Roche Diagnostics, Seoul, Korea) and a cobas e801 analyzer. Serum TRAb concentrations were measured by an Elecsys^®^ electrochemiluminescence immunoassay (Roche Diagnostics, Seoul, Korea) or a TRAK^™^ Brahms radioimmunoassay (Thermo Scientific, Hennigsdorf, Germany). The normal ranges of fT4, T3, and TSH were 0.7–1.5 ng/dL, 60–160 ng/dL, and 0.4–4.9 uIU/mL, respectively. Normal ranges of TRAb on the electrochemiluminescence immunoassays and radioimmunoassays were 0.3–1.22 IU/L and 0–1.0 IU/L, respectively.

### Pathologic evaluation

All removed thyroid specimens were evaluated by pathologists at Seoul National University Bundang Hospital. If thyroid nodules were present, their pathology and size were evaluated. The maximum width (W), length (L), and height (H) of each thyroid lobe and nodule was measured in centimeters. As the weights of the thyroid glands were not available, thyroid gland volume was estimated using the following equation for ellipsoid volume [17, 18].

$${\uppi }/{6 }* \, ({\text{W}}*{\text{L}}*{\text{H of the right thyroid lobe }} + {\text{ W}}*{\text{L}}*{\text{H of the left thyroid lobe }} + {\text{ W}}*{\text{L}}*{\text{H of the isthmus}},{\text{ if available}})$$

Isthmus was included in the calculation if it was separately dissected. In patients with multiple nodules, the pathologic characteristics and size of the largest thyroid nodule was analyzed.

### Indicators for difficult thyroidectomy

The primary indicators of the difficulty of thyroidectomy in this study were operation time (OP-time) and intraoperative estimated blood loss (EBL), with both parameters obtained from the anesthesia records. OP-time was defined as the interval from the beginning of the first incision to closure of the wound and recorded in 5 min intervals. EBL < 50 mL was recorded as “minimal,” with greater amounts reported in 50 mL intervals. For statistical analysis, non-minimal EBL was defined as EBL ≥ 50 mL.

In evaluating complication rates in the OT and RT groups, hypoparathyroidism was defined as PTH levels < 10 pg/mL after surgery. Hypoparathyroidism was considered transient when the levels returned within 6 months after surgery, and permanent if low levels lasted longer than 6 months. Vocal cord paralysis was defined as voice changes due to recurrent laryngeal nerve injury or decreased movement of vocal cord on postoperative laryngoscope. When the paralysis lasted less than 6 months it was considered transient, and permanent if it persisted for longer than 6 months.

### Statistics

The characteristics of patients in the OT and RT groups were compared using Pearson’s chi-squared test, Wilcoxon rank-sum test, and Fisher’s exact test, as appropriate. The normal distribution of continuous variables was assessed using the Shapiro–Wilk test. Univariable and multivariable regression analyses were also performed, with OP-time (linear) and EBL (logistic) as dependent variables. Covariates for the regression models included sex, body mass index (BMI), thyroid volume, TRAb levels, Graves’ ophthalmopathy, thyroid hormone levels, nodule number, main nodule size, main nodule location, central neck node dissection (CND), antithyroid drug type, antithyroid drug duration, preoperative use of potassium iodide, and smoking status. Factors with p values < 0.05 on univariate analyses were included in multivariable models, with stepwise selection of covariates.

## Results

### Clinicopathologic characteristics

The present study enrolled 85 patients, including 48 who underwent OT and 37 who underwent RT. Patients who underwent RT were significantly younger and had a significantly lower BMI than patients who underwent OT (Table 1). Most patients received methimazole or carbimazole as antithyroid medication, for a median duration of 30.5 months in the OT group and 29.0 months in the RT group. The median TRAb concentrations were 29.4 IU/L in the OT group and 15.2 IU/L in the RT group (p = 0.27). The most common reason for surgery was disease intractability, reported by 33.3% and 43.2% of patients in the OT and RT groups, respectively. Potassium iodide was administered preoperatively to 25.0% and 32.4% of patients in the OT and RT groups, respectively. The median thyroid volumes in the OT and RT groups were 72.4 g and 57.6 g, respectively (p = 0.09; Table 1). The pathologic information is summarized in Supplementary Table 1.

**Table 1:** Clinical characteristics

Characteristic	OT (N = 48)	RT (N = 37)	p value
Sex			
Men	13 (27.1%)	5 (13.5%)	0.13
Women	35 (72.9%)	32 (86.5%)	
Age	45.0 (33.8, 57.3)	34.0 (30.0, 44.0)	0.004
BMI	24.3 (21.8, 26.2)	21.7 (20.4, 25.3)	0.05
Smoker			
Never	36 (75.0%)	24 (64.9%)	0.21
Previous	3 (6.2%)	7 (18.9%)	
Current	9 (18.8%)	6 (16.2%)	
ATD type			
Methimazole/carbimazole	35 (72.9%)	29 (78.4%)	0.31
Propylthiouracil	1 (2.1%)	2 (5.4%)	
Both, separately	8 (16.7%)	6 (16.2%)	
n/a	4 (8.3%)	0 (0.0%)	
ATD duration (months)	30.5 (13.8, 43.0)	29.0 (21.0, 43.0)	0.77
Preoperative RAI treatment			
Never	47 (97.9%)	36 (97.3%)	0.68
Once	0 (0.0%)	1 (2.7%)	
Twice	1 (2.1%)	0 (0.0%)	
fT4 (ng/dL)	1.6 (1.2, 2.1)	1.6 (1.1, 2.4)	0.59
T3 (ng/dL)	183.0 (153.0, 224.0)	179.0 (155.0, 247.5)	0.74
TSH (IU/mL)	0.1 (0.1, 0.2)	0.1 (0.1, 0.1)	0.90
TRAb (IU/L)	29.4 (6.7, 47.7)	15.2 (6.9, 36.2)	0.27
Thyroid volume (g)	72.4 (52.1, 129.2)	57.6 (37.0, 94.4)	0.09
Nodule number	1.0 (0.0, 1.3)	1.0 (0.0, 2.0)	0.12
Main nodule size (cm)	1.7 (0.8, 2.8)	1.0 (0.7, 1.3)	0.03
Operative indication			
Goiter with symptom	11 (22.9%)	4 (10.8%)	0.15
Uncontrolled with ATD/frequent recurrence	16 (33.3%)	16 (43.2%)	0.35
Graves’ opthalmopathy	14 (29.2%)	10 (27.0%)	0.83
Suspicous/biopsy proven malignant nodule	15 (31.3%)	12 (32.4%)	0.91
Preparing for pregnancy	0 (0.0%)	3 (8.1%)	0.08
Medication intolerance	4 (8.3%)	4 (10.8%)	0.72
Patient choice	2 (4.2%)	1 (2.7%)	> 0.99
Other	1 (2.1%)	0 (0.0%)	> 0.99
Preoperative medication			
None	4 (8.3%)	4 (10.8%)	0.70
ATD	39 (81.3%)	31 (83.8%)	0.76
Potassium iodide	12 (25.0%)	12 (32.4%)	0.09
Beta blocker	13 (27.1%)	7 (18.9%)	0.38
Steroid	2 (4.2%)	0 (0.0%)	> 0.99
Lithium	1 (2.1%)	0 (0.0%)	> 0.99

Continuous variables are shown as median (interquartile range), and categorical variables are shown as number (percentage)

*OT* open thyroidectomy, *RT* robotic thyroidectomy, *BMI* body mass index, *ATD* antithyroid drug, *n/a* not available, *RAI* radioactive iodine, *fT4* free thyroxine, *T3* triiodothyronine, *TSH* thyroid stimulating hormone, *TRAb* thyroid stimulating hormone receptor antibody, *ATD* antithyroid drug

### Operative outcomes

Operative outcomes were generally comparable in the two groups, except that OP-time was significantly longer in the RT than in the OT group (155.0 vs. 85.0 min, respectively; p < 0.001). In contrast, EBL were comparable between OT and RT groups (Table 2). Postoperative complications, including rates of hypoparathyroidism, vocal cord palsy, and seroma, did not differ significantly in the two groups (Table 2). Two patients in the RT group received an additional 2 cm mini-incision at the upper neck for ligation of the superior thyroid vessels.

**Table 2:** Operative outcomes

Characteristic	OT (N = 48)	RT (N = 37)	p value
Central neck node dissection			
None	43 (89.6%)	27 (73.0%)	0.11
Right	0 (0.0%)	3 (8.3%)	
Left	3 (6.3%)	5 (13.5%)	
Both	2 (4.2%)	2 (5.6%)	
Operation time (min)	85.0 (75.0, 101.3)	155.0 (125.0, 175.0)	< 0.001
Estimated blood loss (ml)			
Minimal (< 50 ml)	34 (70.8%)	30 (81.1%)	0.28
Non-minimal (≥ 50 ml)^a^	14 (29.1%)	7 (18.9%)	
Additional cervical mini-incision^b^			
No	n/a	35 (94.6%)	n/a
Yes	n/a	2 (5.4%)	
Hospital stay (days)	4 (4, 5)	4 (4, 5)	0.88
Reoperation for bleeding control	1 (2.1%)	0 (0.0%)	> 0.99
Hypoparathyroidism			
Transient	11 (22.9%)	16 (43.2%)	0.12
Permanent	3 (6.3%)	2 (5.4%)	
Vocal cord palsy			
Transient	3 (6.3%)	0 (0.0%)	0.25
Permanent	0 (0.0%)	0 (0.0%)	
Seroma^c^	6 (12.5%)	1 (2.7%)	0.13

Continuous variables are shown as median (interquartile range), and categorical variables are shown as number (percentage)

*OT* open thyroidectomy, *RT* robotic thyroidectomy, *PTC* papillary thyroid cancer, *n/a* not available

^a^Median (interquartile range) values of non-minimal estimated blood loss (ml) in OT and RT groups are 100.0 (100.0, 187.5) and 100.0 (50.0, 425), respectively

^b^Two patients underwent additional cervical 2 cm mini-incision during robotic thyroidectomy for ligation of the superior thyroid vessels

^c^Resolved without intervention

### Predictors of operation time

Univariable and multivariable linear regression analyses were performed to find factors affecting OP-time in OT and RT patients (Table 3). In the OT group, greater thyroid volume was the only factor that prolonged OP-time (β = 0.19, 95% confidence interval [CI] 0.13–0.24; p < 0.001). In the RT group, univariable analyses revealed that greater thyroid volume, higher TRAb, T3, and fT4 levels, main nodules located at the isthmus, and bilateral CND were associated with prolonged OP-time. Multivariable analysis showed that factors independently associated with longer OP-time in RT were greater thyroid volume, higher T3 and fT4 levels, and bilateral CND. By contrast, TRAb was not independently associated with OP-time in patients undergoing RT (Table 3).

**Table 3:** Predictors of longer operation time

Characteristic	OT			RT					
	Univariable models			Univariable models			Mutivariable model		
	Beta	95% CI	p value	Beta	95% CI	p value	Beta	95% CI	p value
Sex									
Male	–	–		–	–				
Female	− 11	− 26, 3.8	0.15	13	− 35, 61	0.59			
BMI	0.40	− 1.8, 2.6	0.72	− 0.13	− 3.6, 3.4	0.94			
Thyroid volume	0.19	0.13, 0.24	< 0.001	0.43	0.17, 0.68	0.003	0.68	0.11, 1.2	0.04
TRAb	0.00	− 0.05, 0.05	0.95	0.73	0.39, 1.1	< 0.001	0.36	− 0.29, 1.0	0.30
Graves’ opthalmopathy	8.9	− 5.9, 24	0.24	11	− 26, 48	0.57			
T3	0.05	− 0.02, 0.12	0.16	0.19	0.03, 0.35	0.02	− 0.58	− 0.93, -0.22	0.008
fT4	− 3.0	− 8.6, 2.6	0.30	14	2.5, 25	0.02	28	3.2, 53	0.05
TSH	0.53	− 0.50, 1.6	0.32	− 2.7	− 8.0, 2.6	0.32			
Nodule number	0.59	− 4.7, 5.9	0.83	5.7	− 3.2, 15	0.22			
Main nodule size	1.4	− 4.4, 7.3	0.63	13	− 5.1, 32	0.17			
Main nodule site									
Upper	–	–		–	–		–	–	
Middle	− 3.8	− 56, 49	0.89	40	− 9.2, 89	0.13	20	− 10, 50	0.22
Lower	− 1.0	− 60, 58	0.97	1.0	− 79, 81	0.98	-37	− 88, 14	0.18
Isthmus	− 35	− 107, 37	0.35	136	31, 241	0.02	52	− 92, 197	0.49
Central neck node dissection									
None	–	–		–	–		–	–	
Right	− 13	− 41, 15	0.38	− 25	− 81, 31	0.39	6.0	− 33, 45	0.77
Left	1.3	− 33, 36	0.94	− 24	− 69, 21	0.29	− 15	− 50, 19	0.40
Both				81	13, 149	0.03	53	8.8, 98	0.04
ATD type	–	–							
Methimazole/carbimazole	1.7	− 46, 49	0.95	–	–				
Propylthiouracil	− 9.6	− 28, 8.9	0.31	− 12	− 84, 61	0.76			
Both, separately	− 16	− 41, 9.0	0.22	28	− 16, 73	0.22			
ATD duration	− 0.16	− 0.59, 0.27	0.47	1.2	0.03, 2.3	0.05	0.00	− 0.98, 0.98	> 0.99
Potassium iodide	− 5.1	− 21, 11	0.53	− 16	− 50, 19	0.39			
Smoker									
Never	–	–		–	–				
Previous	21	− 6.9, 49	0.15	− 15	− 58, 29	0.51			
Current	6.5	− 11, 24	0.46	− 7.9	− 54, 38	0.74			

*OT* open thyroidectomy, *RT* robotic thyroidectomy, *BMI* body mass index, *T3* triiodothyronine, *fT4* free thyroxine, *TSH* thyroid stimulating hormone, *CI* confidence interval, *TRAb* Thyroid stimulating hormone receptor antibody, *PTC* papillary thyroid cancer, *ATD* antithyroid drug

### Predictors of intraoperative estimated blood loss

Logistic analyses with the same covariates were performed to identify factors associated with intraoperative blood loss (Table 4). In the OT group, higher thyroid volume was marginally associated with non-minimal EBL (β = 0.01, 95% CI 0.00–0.01; p = 0.07). In the RT group, univariable analyses showed that higher TRAb and thyroid hormone levels (T3, fT4) were associated with non-minimal EBL. Multivariable analysis found that only TRAb (β = 0.05, 95% CI 0.01–0.10; p = 0.04) was independently associated with EBL in patients undergoing RT.

**Table 4:** Predictors of increased intraoperative estimated blood loss

Characteristic	OT			RT					
	Univariable models			Univariable models			Mutivariable model		
	Beta	95% CI	p value	Beta	95% CI	p value	Beta	95% CI	p value
Sex									
Male	–	–		–	–				
Female	− 1.1	− 2.4, 0.29	0.12	− 1.3	− 3.3, 0.92	0.22			
BMI	0.14	− 0.07, 0.35	0.20	− 0.11	− 0.37, 0.09	0.36			
Thyroid volume	0.01	0.00, 0.01	0.07	0.01	0.00, 0.02	0.15			
TRAb	0.00	− 0.02, 0.00	0.50	0.04	0.01, 0.07	0.01	0.05	0.01, 0.10	0.04
Graves’ opthalmopathy	− 0.04	− 1.5, 1.3	0.95	0.90	− 0.90, 2.6	0.30			
T3	0.00	− 0.01, 0.01	0.82	0.02	0.00, 0.03	0.02	0.02	0.00, 0.05	0.14
fT4	− 0.45	− 1.3, 0.16	0.23	0.66	0.07, 1.5	0.05	− 0.01	− 2.3, 2.0	> 0.99
TSH	0.01	− 0.11, 0.11	0.81	− 11	− 43, − 2.6	0.43			
Nodule number	− 0.06	− 0.63, 0.41	0.81	0.03	− 0.47, 0.46	0.88			
Main nodule size	− 0.32	− 1.1, 0.22	0.31	0.49	− 0.57, 1.5	0.26			
Main nodule site									
Upper	–	–		–	–				
Middle	17	− 789, n/a	> 0.99	18	− 1,649, n/a	> 0.99			
Lower	17	− 662, n/a	> 0.99	0.00		> 0.99			
Isthmus	35	− 836, n/a	> 0.99	41	− 3,795, n/a	> 0.99			
Central neck node dissection									
None	–	–		–	–				
Right	1.6	− 0.79, 4.8	0.20	− 17		> 0.99			
Left	− 16		> 0.99	− 17		> 0.99			
Both				1.3	− 2.1, 4.6	0.40			
ATD type	–	–							
Methimazole/Carbimazole	− 16		> 0.99	–	–				
Propylthiouracil	− 0.32	− 2.3, 1.3	0.72	− 16		> 0.99			
Both, separately	0.00	− 0.04, 0.04	> 0.99	1.8	− 0.11, 3.8	0.06			
ATD duration	− 1.1	− 2.4, 0.29	0.12	0.07	0.00, 0.16	0.07			
Potassium iodide	− 0.92	− 2.9, 0.61	0.28	− 0.22	− 2.3, 1.5	0.81			
Smoker									
Never	–	–		–	–				
Previous	0.41	− 2.7, 2.9	0.75	− 0.18	− 3.3, 2.0	0.88			
Current	0.88	− 0.69, 2.4	0.26	0.92	− 1.3, 2.9	0.37			

*OT* open thyroidectomy, *RT* robotic thyroidectomy, *T3* triiodothyronine, *fT4* free thyroxine, *TSH* thyroid stimulating hormone, *CI* confidence interval, *TRAb* thyroid stimulating hormone receptor antibody, *PTC* papillary thyroid cancer, *ATD* antithyroid drug, *n/a* not available

### Comparisons of thyroid volume and TRAb by blood loss

**Fig. 1:**
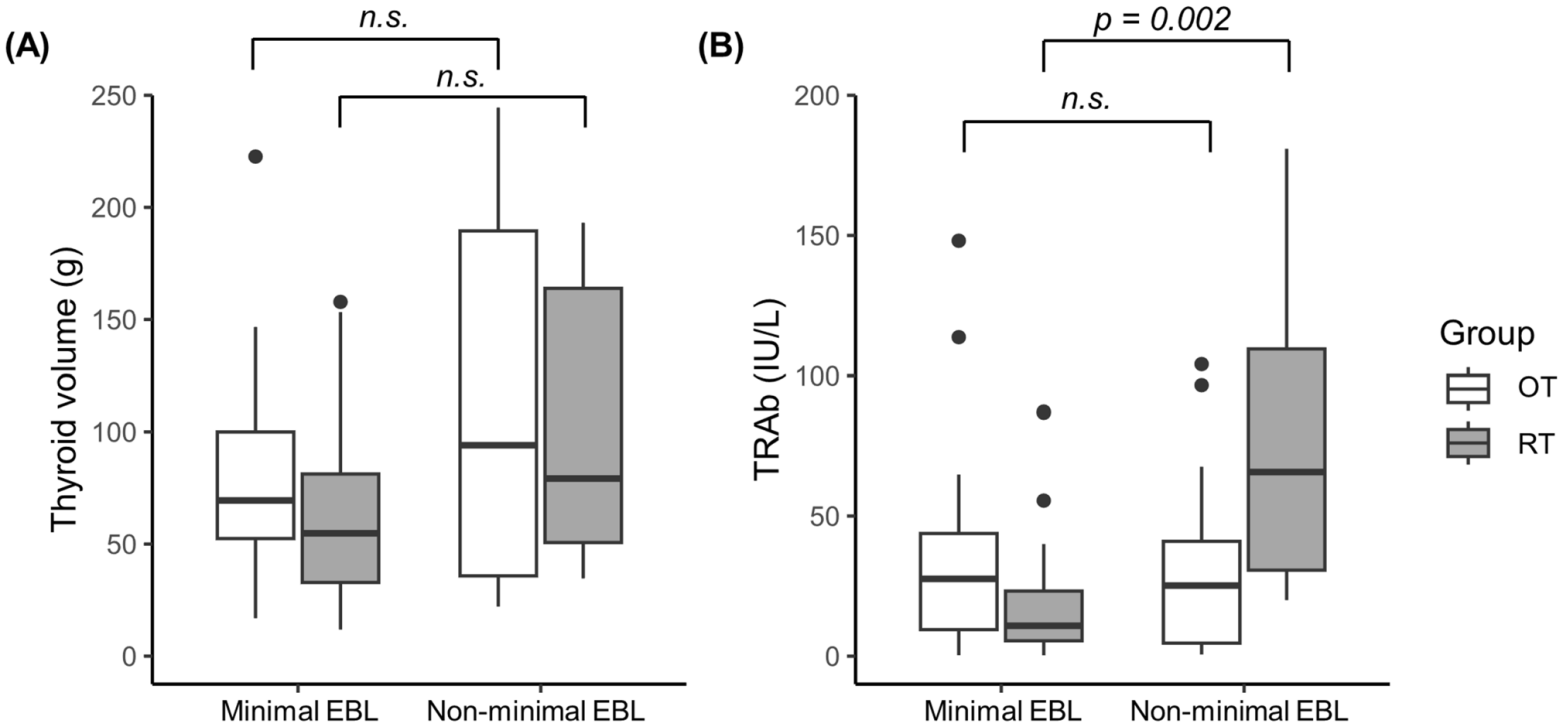
Comparisons of (**A**) Thyroid volume and (**B**) TSH receptor antibody by EBL. *OT* open thyroidectomy, *RT* robotic thyroidectomy, *EBL* estimated blood loss, *TRAb* thyroid stimulating hormone receptor antibody, *n.s* not significant

To investigate differences in thyroid volume and TRAb levels between patients with minimal and non-minimal EBL, Wilcoxon rank-sum tests were conducted in both OT and RT groups (Fig. 1). No significant differences in thyroid volume were observed between minimal and non-minimal EBL patients in either OT or RT groups. However, TRAb levels were significantly higher in non-minimal EBL patients who underwent RT compared to those with minimal EBL (p = 0.002). Conversely, TRAb levels were comparable within the OT group irrespective of degree of EBL.

### Comparison of operative outcomes by TRAb in robotic thyroidectomy

To further explore the role of TRAb levels, we conducted a subgroup analysis of patients who underwent RT. We divided the patients into two groups based on their median TRAb level (15.2 IU/L). While the overall complication rates were not significantly different between the groups (Supplementary Table 2), patients with higher TRAb levels (≥ 15.2 IU/L) had significantly longer OP-time compared to the lower TRAb group (p = 0.02). Significantly, only 63% of patients with higher TRAb levels had minimal EBL, compared to 100% of patients in the lower TRAb group (p = 0.008).

## Discussion

This study showed that factors predicting difficult thyroidectomy for GD patients differed by surgical approaches. Thyroid volume was the only factor that was associated with difficult thyroidectomy in patients who underwent OT. In patients who underwent RT, thyroid volume was associated with OP-time, while only TRAb levels significantly predicted increased EBL. In line with previous findings, preoperative thyroid hormone levels and bilateral CND were also associated with difficult thyroidectomy in the RT group [19, 20], whereas other factors, such as duration of antithyroid drug treatment, preoperative administration of potassium iodide, and smoking status, were not associated.

Because surgical techniques are dependent on operative approaches, factors predictive of difficult thyroidectomy should be analyzed separately for each approach. Previous studies have shown that factors that reduce bleeding in GD patients include preoperative iodine administration and longer antithyroid drug treatment, while thyroid volume increased risks of bleeding [1, 21–23]. These studies, however, evaluated GD patients who underwent conventional OT. To our knowledge, the present study is the first to compare factors predictive of difficult thyroidectomy in GD patients by operation types.

As TRAb plays a major role in the pathophysiology of GD, it is strongly associated with disease severity and prognosis [6]. No study, however, has analyzed the association of TRAb with operative outcomes, except one study which reported that TRAb did not affect intraoperative blood loss in GD patients who underwent OT [1]. Our study was in line with their findings, as TRAb did not affect EBL in OT patients. By contrast, TRAb concentration was a significant predictor of increased EBL in patients who underwent RT. This association may be due to the role of TRAb in increasing thyroid vascularity [5, 24]. Both thyroid vascularity and peak systolic velocity of the superior thyroid vessels are associated with TRAb titer [4, 25, 26], as TRAb stimulates TSH receptors which have a proangiogenic role. Because TSH receptor is expressed on human dermal microvascular endothelial cells, TSH stimulates angiogenesis by promoting capillary network formation [27]. TSH was also found to increase angiogenic markers, such as *VEGF*, *angiopoietins*, and *Tie-2*, in vitro [28, 29]. Therefore, it can be hypothesized that GD patients with elevated TRAb levels have hypervascular thyroid glands, increasing EBL during thyroidectomy.

TRAb increased EBL in patients who underwent RT, but not OT, suggesting that differences in operative techniques and procedures may influence EBL. The initial stage of OT consists of superior pole dissection with early ligation of the superior thyroid vessels. In BABA RT, however, thyroidectomy is performed in a caudal to cranial direction, ligating the superior thyroid vessels at last. Because higher TRAb levels are associated with increased blood flow through the superior thyroid vessels [25, 26], patients with higher TRAb would be exposed to greater vascular flow throughout the operation. Moreover, traction of the thyroid gland with engorged vessels often leads to bleeding, as the vein may be torn during handling of the thyroid gland. This is especially difficult in RT, as handling the engorged vessels with metallic robotic arms, which lack tactile sense, may lead to easier touch-bleeding. In OT, however, the use of fingers and gauzes for traction of the thyroid gland may prevent bleeding from the thyroid surface.

Immediate control of severe bleeding may take longer during RT than OT, as blood accumulation can impede the camera’s view. Moreover, it is difficult to control bleeding from the cut-end of the superior thyroid vessels during BABA RT, as the robotic arms may be unable to reach deeper into the bleeding focus. Two patients in the present study who underwent RT experienced superior thyroid vein bleeding, requiring a 2 cm mini cervical incision at the upper neck to ligate the superior thyroid vein. Both patients had high TRAb levels, 143.3 and 180.9 IU/L, respectively, and severely engorged thyroid vessels.

The present study had several limitations. First, EBL is a crude estimate of intraoperative blood loss, resulting in possible information bias. Because this was a preliminary retrospective study, further prospective studies are needed using specific determinations of blood loss to assess its association with TRAb. Second, selection bias could not be avoided, as this study was retrospective in design and included a small number of study subjects from a single center, with all undergoing surgery by a single surgeon. Third, the results do not represent all types of robotic approaches, as all RT patients in this study underwent BABA RT. Because other types of RT have different operative procedures than BABA RT, further studies are needed to identify individual factors for difficult thyroidectomy in each approach. Fourth, TRAb was not measured at the same time relative to surgery in all patients, with measurements obtained within 6 months prior to surgery used, leading to possible information bias. Finally, while autoantibodies can both stimulate and block TSH receptors, the immunoassays and radioassays used in this study could not differentiate the activities of the TRAb [30]. Cell-based bioassays are appropriate for this differentiation, but these assays are not readily available in clinical practice [30]. However, since most GD patients underwent thyroidectomy due to uncontrolled disease caused by overstimulation by TRAb, using clinical TRAb results as a guide to predict increased EBL may be feasible.

In conclusion, caution should be exercised in performing BABA RT on GD patients with high level of TRAb, regardless of thyroid size. Preoperative preparation of intravascular lines and suction devices, and readiness to make additional incisions may be helpful in performing BABA RT on GD patients with high TRAb. Alternatively, conventional OT may be more feasible in these patients. Other factors, such as bilateral CND, high thyroid hormone levels, and high thyroid volume have been associated with longer OP-time or higher EBL, as well. Prospective studies in large numbers of patients are needed to support these findings.

## Supplementary Information

Below is the link to the electronic supplementary material.

Supplementary file1 (DOCX 21 KB)

## Data Availability

The dataset for the current study is available from the corresponding author upon reasonable request.
