# Supplementary material for: TSH receptor antibody as a predictor of difficult robotic thyroidectomy in patients with Graves’ disease
Source: J Robot Surg. 2024 Mar 4;18(1):108. doi: 10.1007/s11701-024-01869-y (PMC10912279; doi:10.1007/s11701-024-01869-y)
Supplement: Supplementary file 1 — Supplementary file1 (DOCX 21 KB) [file 11701_2024_1869_MOESM1_ESM.docx]

**TSH Receptor Antibody as a Predictor of Difficult Robotic Thyroidectomy in Patients with Graves' Disease**

*Journal of Robotic Surgery*

Ja Kyung Lee, M.D., M.S.^1^, Yoon Kong, M.D., M.S.^1^, Jae Bong Choi, M.D., M.S.^1^, Woochul Kim, M.D.^2^, Hyeong Won Yu, M.D., Ph.D.^1,3^, Su-jin Kim, M.D., Ph.D.^2,3^, Young Jun Chai, M.D., Ph.D.^3,4^, June Young Choi, M.D., Ph.D.^1,3,^*, Kyu Eun Lee, M.D., Ph.D.^2,3^

^1^Department of Surgery, Seoul National University Bundang Hospital, Seongnam-si, Korea

^2^Department of Surgery, Seoul National University Hospital, Seoul, Korea

^3^Department of Surgery, Seoul National University College of Medicine, Seoul, Korea

^4^Department of Surgery, Seoul Metropolitan Government Seoul National University Boramae Medical Center, Seoul, Korea

* Corresponding author e-mail: may11@snu.ac.kr

**Supplementary Table 1. Pathologic information**

| **Characteristic** | **OT** (N = 48) | **RT** (N = 37) | **p-value** |
| --- | --- | --- | --- |
| Main nodule site |  |  | 0.30 |
| Upper | 1 (3.8%) | 5 (20.8%) |  |
| Middle | 21 (80.8%) | 16 (66.7%) |  |
| Lower | 3 (11.5%) | 2 (8.3%) |  |
| Isthmus | 1 (3.8%) | 1 (4.2%) |  |
| Main nodule pathology |  |  | 0.48 |
| Classic PTC | 6 (23.1%) | 6 (25.0%) |  |
| Tall cell variant PTC | 1 (3.8%) | 4 (16.7%) |  |
| Infiltrative follicular PTC | 1 (3.8%) | 0 (0.0%) |  |
| Nodular hyperplasia | 6 (23.1%) | 1 (4.2%) |  |
| Cellular adenomatoid nodule | 2 (7.7%) | 3 (12.5%) |  |
| Follicular adenoma | 2 (7.7%) | 1 (4.2%) |  |
| NIFTP | 1 (3.8%) | 1 (4.2%) |  |
| Cyst | 1 (3.8%) | 1 (4.2%) |  |
| Hurthle cell adenoma | 1 (3.8%) | 0 (0.0%) |  |
| n/a | 5 (19.2%) | 7 (29.2%) |  |

Variables are shown as number (percentage).

Abbreviations: *OT* open thyroidectomy; *RT* robotic thyroidectomy; *PTC* papillary thyroid cancer; *NIFTP* Noninvasive follicular thyroid neoplasm with papillary-like nuclear features; *n/a* not available

**Supplementary Table 2. Comparison of operative outcomes by TRAb levels in robot thyroidectomy patients**

| **Characteristic** | **TRAb <15.2 IU/L** (N = 18) | **TRAb ≥15.2 IU/L** (N = 19) | **p-value** |
| --- | --- | --- | --- |
| Operation time (min) | 140 (118, 165) | 170 (148, 198) | 0.02 |
| Estimated blood loss (ml) |  |  | 0.008 |
| Minimal (<50 ml) | 18 (100%) | 12 (63%) |  |
| Non-minimal (≥50 ml) | 0 (0%) | 7 (37%) |  |
| Additional cervical mini-incision^a^ | 0 (0%) | 2 (11%) | 0.49 |
| Hospital stay (days) | 4 (4, 5) | 5 (4, 5) | 0.17 |
| Complication |  |  | 0.87 |
| No | 9 (50%) | 9 (47%) |  |
| Yes | 9 (50%) | 10 (53%) |  |
| Hypoparathyroidism |  |  | 0.87 |
| No | 10 (56%) | 9 (47%) |  |
| Transient | 7 (39%) | 9 (47%) |  |
| Permanent | 1 (5.6%) | 1 (5.3%) |  |
| Vocal cord palsy |  |  | >0.99 |
| No | 18 (100%) | 19 (100%) |  |
| Seroma | 1 (5.6%) | 0 (0%) | 0.49 |

Continuous variables are shown as median (interquartile range), and categorical variables are shown as number (percentage).

Abbreviations: *TRAb* thyroid stimulating hormone receptor antibody

^a^Two patients underwent additional cervical 2 cm mini-incision during robotic thyroidectomy for ligation of the superior thyroid vessels
